# The Curcumin Analog Da0324 Inhibits the Proliferation of Gastric Cancer Cells *via* HOTAIRM1/miR-29b-1-5p/PHLPP1 Axis

**DOI:** 10.7150/jca.69970

**Published:** 2022-05-16

**Authors:** Fanfan Xu, Mengxia Chen, Hao Chen, Nan Wu, Qinqin Qi, Xiujiao Jiang, Daoquan Fang, Qian Feng, Rong Jin, Lei Jiang

**Affiliations:** 1 Department of Gastroenterology, the First Affiliated Hospital of Wenzhou Medical University, Wenzhou 325000, China.; 2 Department of Epidemiology, the First Affiliated Hospital of Wenzhou Medical University, Wenzhou 325000, China.; 3 Central Laboratory, the First Affiliated Hospital of Wenzhou Medical University, Wenzhou 325000, China.

**Keywords:** curcumin analog, Da0324, gastric cancer, HOTAIRM1, miR-29b-1-5p, PHLPP1

## Abstract

**Background:** Our previous study has shown that Da0324, a curcumin analog, exhibited significantly improved stability and antitumor activity. However, the molecular mechanisms of action of Da0324 remain poorly understood. Long non-coding RNA (lncRNA) has been shown to play a key role in tumor progression. Here, we aim to investigate the molecular mechanisms underlying the anti-cancer activity of Da0324 by regulating the lncRNA HOTAIRM1.

**Methods:** Gastric cancer cell lines were treated with Da0324 and/or transfected with lentiviral vector expressing HOTAIRM1 shRNA, and/or miR-29b-1-5p mimics and/or small interference RNA (siRNA) against PHLPP1, or HOTAIRM1 siRNA or lentiviral vector expressing HOTAIRM1, as needed. The expression of HOTAIRM1, miR-29b-1-5p and PHLPP1 in GC cells was determined by Real-Time PCR. Cell growth was examined by CCK-8 assay and colony formation assay *in vitro*. The targeted relationship between HOTAIRM1 and miR-29b-1-5p was verified by luciferase reporter gene assay. PHLPP1 protein expression was examined by Western blotting.

**Results:** Da0324 increased the expression of HOTAIRM1 in GC cells. HOTAIRM1 expression was significantly down-regulated in GC tissues, and the low expression of HOTAIRM1 was associated with the shorter survival rate of GC patients based on the TCGA database. Knockdown of HOTAIRM1 promoted GC cell proliferation whereas overexpression of HOTAIRM1 inhibited GC cell proliferation as demonstrated by CCK-8 and colony formation assays. Moreover, knockdown of HOTAIRM1 reversed the Da0324-mediated growth inhibition of GC cells. Furthermore, HOTAIRM1 acted as a sponge for miR-29b-1-5p and PHLPP1 is regulated by the HOTAIRM1/miR-29b-1-5p axis in GC cells. Overexpression of miR-29b-1-5p or knockdown of PHLPP1 reversed the ability of Da0324 to inhibit the growth of GC cells.

**Conclusions:** Our data suggest that Da0324 exerts antitumor activity by regulating HOTAIRM1/miR-29b-1-5p/PHLPP1 axis in GC cells, and provide new insights into the anti-cancer mechanism of Da0324.

## Introduction

Gastric cancer (GC) is one of the most common cancers in the world 1. In China, it is the second most common cancer and the third leading cause of cancer-related deaths 1-3. Because of the lack of specific symptoms, most patients with GC are diagnosed in the advanced stage at the time of diagnosis, resulting in poor prognosis 4-6. Although advances in surgical treatment and chemotherapy have improved GC outcomes in recent decades, the prognosis is still unsatisfactory 7-9. Therefore, further efforts are needed to search for new alternative therapies and improve the efficacy of comprehensive treatment strategies in GC.

Since the severe side effects of chemotherapy affects cancer treatment 10, 11, more and more researches are focusing on the anti-cancer properties of natural products, which may lead to the discovery of drugs that are more effective in cancer treatment and have fewer side effects 12. As an active component extracted from the spice turmeric, curcumin is widely used as a colorant and spice in food 13. Curcumin has anti-oxidant, anti-inflammatory, anti-apoptotic and anti-cancer properties, so more and more researchers are paying attention to it 14, 15. Due to the shortcomings of using curcumin as an anticancer agent, such as its poor water solubility, which limits its oral bioavailability, scientists have developed a variety of strategies to overcome the existing shortcomings 16. One strategy to address the poor water solubility is to use nanoparticle technology to improve bioavailability and cellular uptake 17-19. In our preceding studies, the strategy to overcome the defects was to synthesize the curcumin analog Da0324. Our previous study showed that Da0324 is more stable than curcumin both in phosphate buffer and culture medium 2. The half-life of elimination of Da0324 in culture medium reaches 2.6 h which is longer than that of curcumin (0.9 h). Both of Da0324 and curcumin reveal toxicity to the GC cells. But the half maximal inhibitory concentration (IC50) value at 48 h is lower than 5 μM in most GC cells which mean the toxicity of Da0324 is much stronger than curcumin 2. Da0324 exhibited excellent target selectivity by inhibiting the activation of NF-κB in GC cells and had low toxicity to normal gastric mucosal epithelial cells 2. Da0324 also activated P53 by down-regulating LINC01021 to exert anti-tumor activity against GC 20. Nevertheless, there are still knowledge gaps in understanding the molecular mechanisms involved in the cytotoxicity of Da0324 against GC. Therefore, it is very important to investigate the molecular mechanism of inhibition of gastric cancer by Da0324.

To date, there is increasing evidence that non-coding RNAs (ncRNAs) are involved as important regulators in various physiological and pathological cellular processes 21, 22. Long non-coding RNA (lncRNA) is a ribonucleotide chain, a group of ncRNAs longer than 200 nucleotides 23. There is ample evidence that lncRNA plays an important role in regulating cancer cell proliferation, apoptosis and metastasis 24, 25. One of the functional mechanisms of lncRNAs is to act as competitive endogenous RNAs (ceRNAs), which compete for microRNAs (miRNAs) binding to increase the expression of miRNAs-targeted mRNAs 26. LncRNA HOTAIRM1 has been reported to play an important role in the progression of cancer 27, 28. Li et al. reported that lncRNA HOTAIRM1 functions as ceRNA of miR-107 and regulates proliferation and invasion in papillary thyroid cancer 27. A recent study also indicated that HOTAIRM1 was downregulated in GC. It regulated the development of GC through miR-17-5p, while upregulation of HOTAIRM1 inhibited the proliferation and migration of GC cells 28.

Here, we aimed to investigate the molecular mechanisms underlying the anti-cancer effect of Da0324 through regulation of HOTAIRM1. Our results revealed that Da0324 treatment upregulated HOTAIRM1, which increased PHLPP1 expression by sponging miR-29b-1-5p in GC cells.

## Materials and methods

### Cell culture

A normal human gastric epithelial cell line (GES-1), a human embryonic kidney 293T cell line (HEK-293T), and human gastric cancer cell lines (AGS and KATO III) were purchased from the Cell Bank of the Chinese Academy of Sciences (Shanghai, China). BGC823 and SGC7901 gastric cancer cell lines were obtained from the China Center for Type Culture Collection (Wuhan, China). AGS cells were grown in F-12K medium (Thermo Fisher Scientific, MA, USA), HEK-293T cells were cultured in Dulbecco modified Eagle medium (DMEM) (Gibco, New York, USA) and the other cell lines were cultured in RPMI-1640 medium (Gibco). All media were supplemented with 10% fetal calf serum (Sigma-Aldrich, St Louis, MO, USA), 100 U/ml penicillin and 10 mg/L streptomycin (Gibco). All cells were cultured at 37 °C under a humidified atmosphere with 5% CO_2_.

### High-throughput sequencing of lncRNAs

The high-throughput sequencing assay was carried out as previously described 20.

### Cell transfection

The lentiviral vector expressing short hairpin RNA (shRNA) specific for HOTAIRM1 (sh-HOTAIRM1 target sequence: 5'-AGAAACTCCGTGTTACTCA-3') was purchased from Genechem Co. (GeneChem, Shanghai, China). The nonspecific shRNAs were used in the control group (sh-NC). The full-length sequence of HOTAIRM1 was inserted into pLVX-Puro (Clontech Laboratories, USA), using empty vectors as controls. Lentivirus was produced as described previously. Besides, small interference RNA (siRNA) against HOTAIRM1 (si-HOTAIRM1-1 sequence: 5'-AGAAACTCCGTGTTACTCA-3'; si-HOTAIRM1-2 sequence: 5'-GCCAGAAACCAGCCATAGT-3') 29, siRNA against PHLPP1 (5'-GGAAGACGCUGCUUCUGAA-3'), miR-29b-1-5p mimics, and the corresponding negative control (NC) were designed and synthesized by RiboBio Biotech (RiboBio, Guangzhou, China). The transfections (50 nM si-HOTAIRM1, miR-29b-1-5p mimics or si-PHLPP1) were carried out using Lipofectamine 3000 Reagent (Gibco) following the instructions of manufacturer.

### RNA extraction and quantitative real-time PCR (qRT-PCR)

Total RNA from cultured cells was extracted using TRIzol Reagent (Gibco). For lncRNA expression analysis, cDNA was synthesized using the lnRcute lncRNA First-Strand cDNA Synthesis Kit (Tiangen Biotech, Beijing, China). For miRNA, reverse transcription was performed using miRNA First Strand cDNA Synthesis (Stem-loop Method) (Sangon Biotech, Shanghai, China). qRT-PCR was performed using SYBR Premix Ex Taq (TaKaRa, Kyoto, Japan) on CFX96 Real-time PCR system (Bio-Rad Laboratories, USA). The following thermocycling conditions were used for the qPCR: Initial denaturation at 95 °C for 30 sec, followed by 40 cycles: 95 °C for 5 sec and 60 °C for 34 sec. GAPDH and U6 were used as internal controls, respectively. The relative gene expression level was calculated using the 2^-ΔΔCt^ method. The primers used in this study were obtained from Sangon Biotech and are listed as follows, HOTAIRM1-F: 5′-CCCACCGTTCAATGAAAG-3′, HOTAIRM1-R: 5′-GTTTCAAACACCCACATTTC-3′; miR-29b-1-5p-F: 5′-CGGCTGGTTTCATATGGTGG-3′, miR-29b-1-5p-R: 5′-AGTGCAGGGTCCGAGGTATT-3′; GAPDH-F: 5′-GTCAAGGCTGAGAACGGGAA-3′, GAPDH-R: 5′-AAATGAGCCCCAGCCTTCTC-3′; U6-F: 5′-GCTT CGGCAGCACATATACTAAAAT-3′, U6-R: 5′-CGCTTCACGAATTTGCGTGTCAT-3′.

### Subcellular fractionation

The nucleus and cytoplasm were separated using the PARIS™ Kit (Thermo Fisher Scientific). According to the manufacturer's instructions, cells were suspended in cell separation buffer. RNA was then extracted and qRT-PCR analysis was conducted to detect the localization of HOTAIRM1. GAPDH and U6 served as control transcripts for cytoplasmic and nuclear RNA, respectively.

### Cell counting kit-8 (CCK-8) assay

CCK-8 assay (Dojondo Laboratories, Kumamoto, Japan) was used to evaluate cell viability. Cells (5000/well) were seeded in a 96-well plates, 10 μL of CCK-8 solution was added into each well at indicated time point and incubated for 2 h. The absorbance at 450 nm was measured using microplate reader.

### Plate colony-formation assay

The transfected or Da0324-treated cells were cultured in six-well plates at a density of 800 cells/well and incubated at 37 °C for 2 weeks. Then, the cells were fixed with 4% paraformaldehyde, stained with crystal violet, and counted under a microscope.

### Dual-luciferase activity assay

The binding site of miR-29b-1-5p to HOTAIRM1 was predicted using lncRNAMap (http://lncRNAMap.mbc.nctu.edu.tw/) or TargetScan 7.2 (http://www.targetscan.org/), respectively. Luciferase plasmid containing wild-type (pmirGLO-HOTAIRM1-WT) putative miR-29b-1-5p binding sites in the HOTAIRM1 sequence was generated. To detect binding between HOTAIRM1 and miR-29b-1-5p, pmirGLO-HOTAIRM1-WT reporter plasmid was co-transfected with miR‐NC or miR-29b-1-5p mimics into HEK-293T cells using the Lipofectamine 3000 reagent (Thermo Fisher Scientific). After transfection for 48 h, the luciferase activity was evaluated through the luciferase reporter assay kit (Promega, Madison, WI, USA) according to the manufacturer's protocol. Renilla luciferase activity was used as a control.

### Western blotting

Briefly, cells were lysed in RIPA buffer containing phenylmethylsulfonyl fluoride, phosphatase inhibitor and protease inhibitor to obtain total protein. Subsequently, the proteins were separated by sodium dodecyl sulfate-polyacrylamide gel electrophoresis (SDS-PAGE) and shifted to polyvinylidene fluoride (PVDF) membranes. The membranes were blocked with 5% nonfat milk for 2 h and then probed with primary antibody against PHLPP1 (1:1000, Affinity, Shanghai, China) and GAPDH (1:1000, Cell Signaling Technology, USA) dilution incubated at 4 °C overnight. GAPDH was used as an internal control. The membranes were incubated with HRP-conjugated anti-rabbit secondary antibody (1:5000, Abcam, USA) for 1 h at room temperature. Finally, the results were analyzed using the ECL reagent (Thermo Fisher Scientific) and visualized using an imaging system (Bio-Rad Laboratories).

### Statistical analysis

Data were analyzed using GraphPad Prism 7.0 (GraphPad Prism, Inc., La Jolla, CA, USA), and the results are presented as mean ± standard deviation (SD). Student's t-test was used to compare differences between two groups. Differences between multiple groups were analyzed with One-way analysis of variance. A value of *P* < 0.05 was considered significant.

## Results

### Da0324 inhibits the growth of GC cells and up-regulates the expression of HOTAIRM1

**Figure 1:**
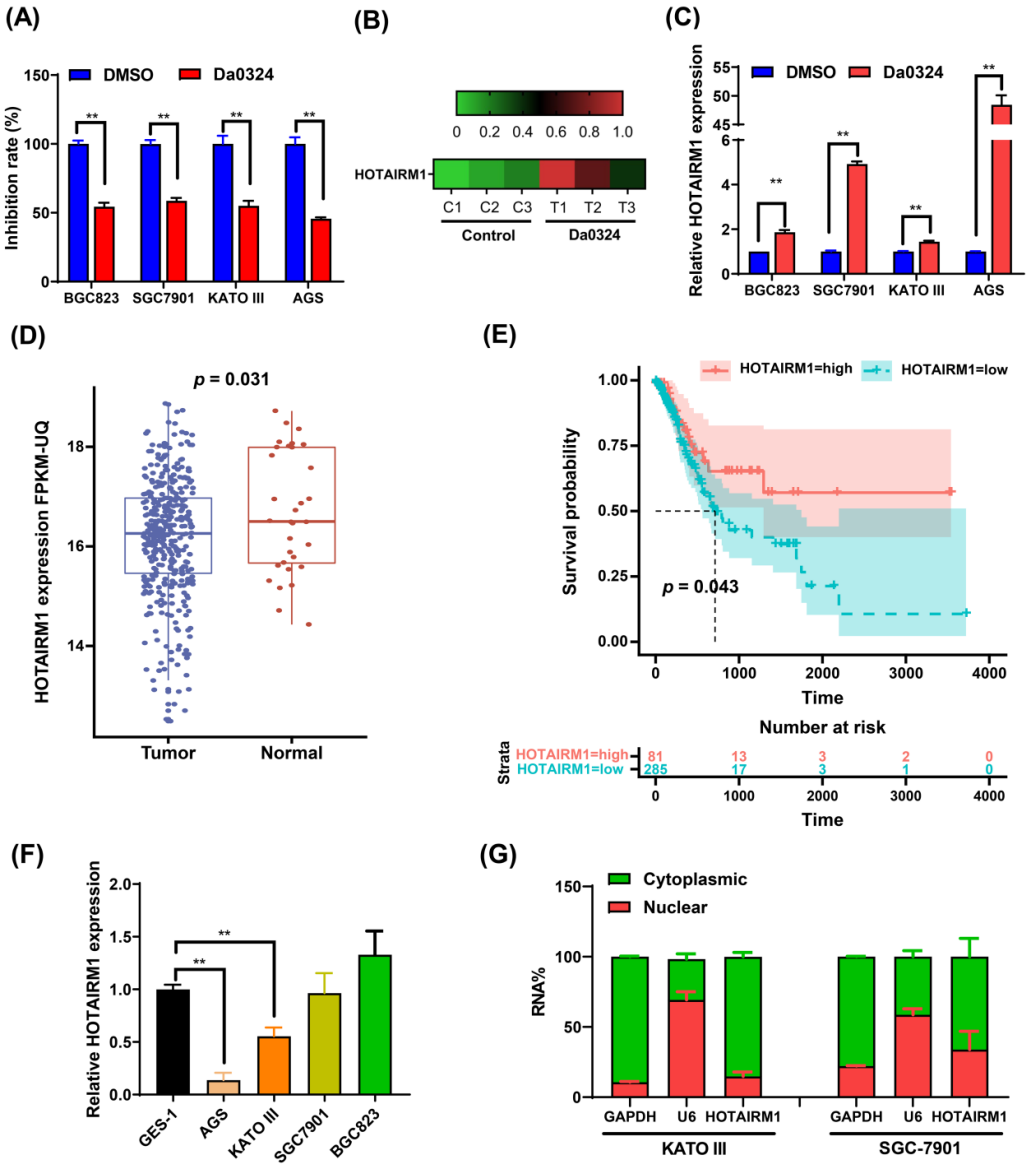
** Da0324 inhibits the growth of gastric cancer cells and up-regulates the expression of HOTAIRM1. (A)** Effects of Da0324 on gastric cancer cell viability. After exposing GC cell lines (BGC823, SGC7901, KATO III and AGS) to 4 µM Da0324 for 48 h, the cytostatic effect was evaluated by CCK-8 assays. **(B)** Heatmap showing the expression levels of HOTAIRM1 in SGC7901 cells treated with Da0324 or control. A high-throughput sequencing assay was performed to screen differentially expressed lncRNAs in SGC7901 cells treated with DMSO or 4 µM Da0324 for 48 h in triplicate. The data of HOTAIRM1 expression levels were analyzed. **(C)** qRT-PCR analysis of HOTAIRM1 expression in GC cells treated with 4 µM Da0324 for 48 h. **(D)** The expression of HOTAIRM1 in normal and gastric cancer tissues based on TCGA data. **(E)** Survival analysis of gastric cancer patients with high and low expression levels of HOTAIRM1. **(F)** qRT-PCR analysis of HOTAIRM1 expression in normal gastric epithelial cell line (GES-1) and GC cell lines (BGC823, SGC7901, KATO III and AGS). **(G)** Relative HOTAIRM1 expression level in the cytoplasm and nucleus of KATO III and SGC7901 cells was determined by qRT-PCR. GAPDH and U6 were used as cytosolic and nuclear loading controls, respectively. All data represent the mean ± SD. Each experiment was performed in triplicate. ^*^, *P* < 0.05; ^**^, *P* < 0.01.

**Figure 1A** shows that Da0324 treatment significantly inhibits GC cell growth. We previously reported that the expression levels of various lncRNAs were detected in Da0324‐treated SGC7901 cells by high-throughput sequencing 20. Interestingly, Da0324 treatment resulted in up-regulation of HOTAIRM1 expression in GC cell lines (**Figure 1B**). The results of qRT-PCR analyses verified that HOTAIRM1 levels increased in response to Da0324 treatment (**Figure 1C**). Using lncRNA expression data from The Cancer Genome Atlas (TCGA), we found that HOTAIRM1 was significantly down-regulated in GC relative to normal samples (**Figure 1D**), and GC patients with low expression of HOTAIRM1 had poorer overall survival than patients with high expression of HOTAIRM1 (**Figure 1E**). We also detected the expression of HOTAIRM1 in 25 pairs of GC tissues and adjacent tissues using qRT-PCR. The expression level of HOTAIRM1 in GC tissues is lower than that in normal tissues (0.98 ± 0.65 *vs.* 1.42 ± 1.28, *p* = 0.107, **Figure S1**), although there is no statistical significance. This might be due to the small sample size. Next, we analyzed the expression of HOTAIRM1 in the normal human gastric epithelial GES-1 cell line and GC cell lines by qRT‐PCR. As revealed in **Figure 1F**, the expression of HOTAIRM1 in KATO III and AGS cells was lower than that of GES-1 cells. Furthermore, in order to better understand the function of HOTAIRM1, we also analyzed its subcellular localization in the nucleus and cytoplasm. Our results revealed that HOTAIRM1 was mainly localized in the cytoplasm of KATO III and SGC7901 cells (**Figure 1G**). This suggests that HOTAIRM1 may have ceRNA function in GC cells.

### HOTAIRM1 knockdown facilitates GC cell proliferation

**Figure 2:**
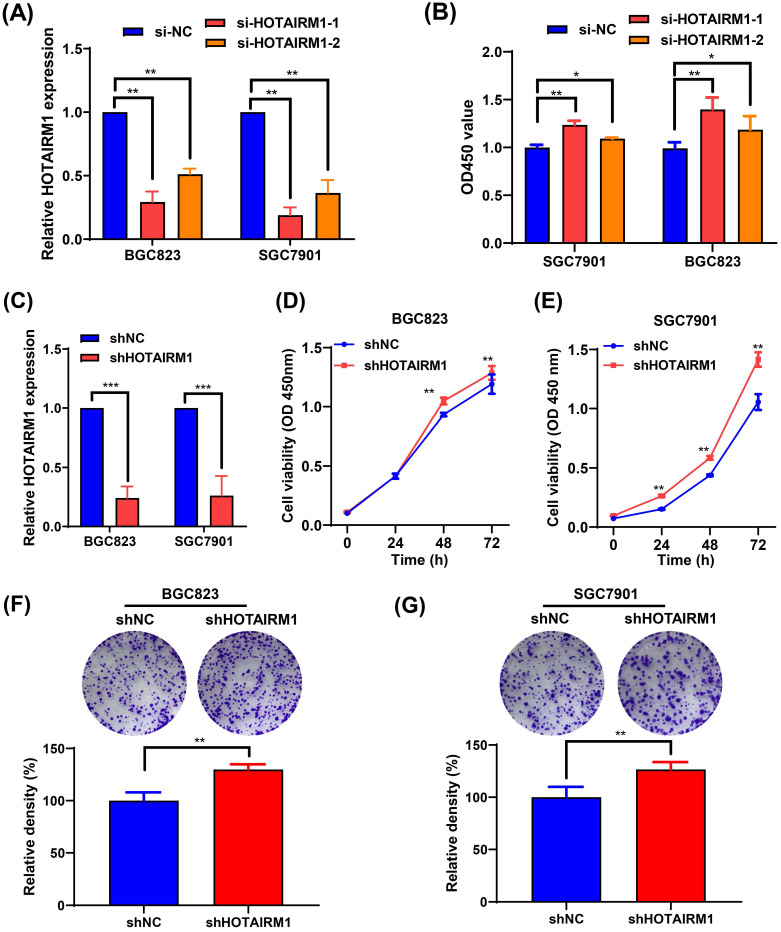
** Knockdown of HOTAIRM1 facilitates GC cell proliferation. (A)** The expression of HOTAIRM1 was determined in BGC823 and SGC7901 cells transfected with HOTAIRM1 siRNAs using qRT-PCR assays. **(B)** Relative cell growth was determined in SGC7901 and BGC823 cells transfected with HOTAIRM1 siRNAs by CCK-8 assays. **(C)** qRT-PCR analysis of HOTAIRM1 expression in BGC823 and SGC7901 cells transfected with shRNA targeting HOTAIRM1 (shHOTAIRM1). **(D-E)** Cell proliferation assay in BGC823 (D) and SGC7901 (E) cells transduced with lenti-shHOTAIRM1. **(F-G)** Colony formation in BGC823 (F) and SGC7901 (G) cells transduced with lenti-shHOTAIRM1 was analyzed with colony formation assays. Representative images of clonogenic assay (upper panel) and quantitative analysis (lower panel). All data represent the mean ± SD. Each experiment was performed in triplicate. ^*^, *P* < 0.05; ^**^, *P* < 0.01.

Because the expression of HOTAIRM1 was relatively high in BGC823 and SGC7901 cells, two specific siRNAs targeting HOTAIRM1 (si-HOTAIRM1-1 and si-HOTAIRM1-2) were designed and transfected into BGC823 and SGC7901 cells to investigate the effects of HOTAIRM1 knockdown on GC cell growth. As shown in **Figure 2A,** si-HOTAIRM1-1 and si-HOTAIRM1-2 effectively knocked down the expression of HOTAIRM1 in GC cells. Next, CCK-8 assays demonstrated that knockdown of HOTAIRM1 increased cell viability in BGC823 and SGC7901 cells (**Figure 2B**). Since si-HOTAIRM1-1 possessed the higher knockdown efficiency, we constructed the lentiviral vector expressing HOTAIRM1 shRNA according to the sequence of si-HOTAIRM1-1. The knockdown efficiency of shHOTAIRM1 was verified in BGC823 and SGC7901 cells by qRT-PCR (**Figure 2C**). The CCK-8 assays showed that the viability of BGC823 and SGC7901 cells was increased after knockdown of HOTAIRM1 (**Figure 2D and 2E**). Similarly, knockdown of HOTAIRM1 promoted cell colony formation in BGC823 and SGC7901 cells (**Figure 2F and 2G**).

### Overexpression of HOTAIRM1 inhibits gastric cancer cell growth

**Figure 3:**
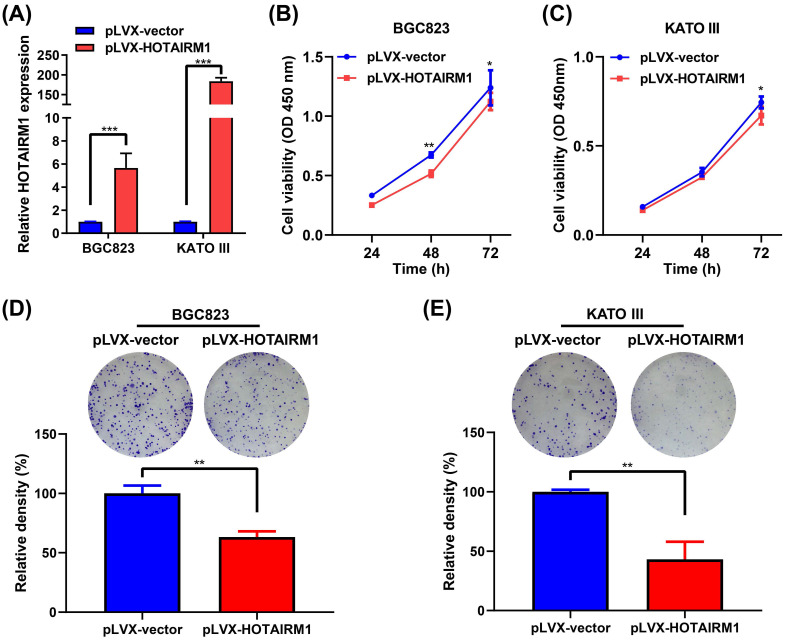
** Overexpression of HOTAIRM1 inhibits proliferation of gastric cancer cells. (A)** qRT-PCR analysis of HOTAIRM1 expression in BGC823 and KATO III cells transduced with lenti-HOTAIRM1. **(B-C)** CCK-8 assays were performed to determine cell viability of BGC823 (B) and KATO III (C) cells after transducion with lenti-HOTAIRM1 or control vector. **(D-E)** Colony-formation assays were performed to determine the effects of HOTAIRM1 on the proliferation of BGC823 (D) and KATO III (E) cells. All experiments were performed in triplicate, and results are presented as mean ± SD. ^*^, *P* < 0.05; ^**^, *P* < 0.01; ^***^, *P* < 0.001.

To investigate the functional role of HOTAIRM1 in GC cells, we performed gain-of-function experiments. qRT-PCR results showed that HOTAIRM1 was overexpressed in BGC823 and KATO III cells transfected with pLVX-HOTAIRM1 (**Figure 3A**). The results of CCK-8 assay showed that overexpression of HOTAIRM1 suppressed the growth of BGC823 and KATO III cells (**Figure 3B and 3C**). As shown in **Figure S2**, overexpression of HOTAIRM1 also inhibited the proliferation of AGS cells. The plate colony formation assay suggested that overexpression of HOTAIRM1 inhibited the colony formation of BGC823 and KATO III cells (**Figure 3D and 3E**). These results demonstrated that HOTAIRM1 could inhibit the proliferation of GC cells. Moreover, we used si-HOTAIRM1-1 to knock down the expression of HOTAIRM1, and co-transfected cells with pLVX-HOTAIRM1 to restore the expression of HOTAIRM1. The result of CCK-8 assay showed that overexpression of HOTAIRM1 reversed the promotion of cell proliferation by si-HOTAIRM1 in BGC832 cells (**Figure S3**).

### Knockdown of HOTAIRM1 alleviate cytotoxicity induced by Da0324 in GC cells

**Figure 4:**
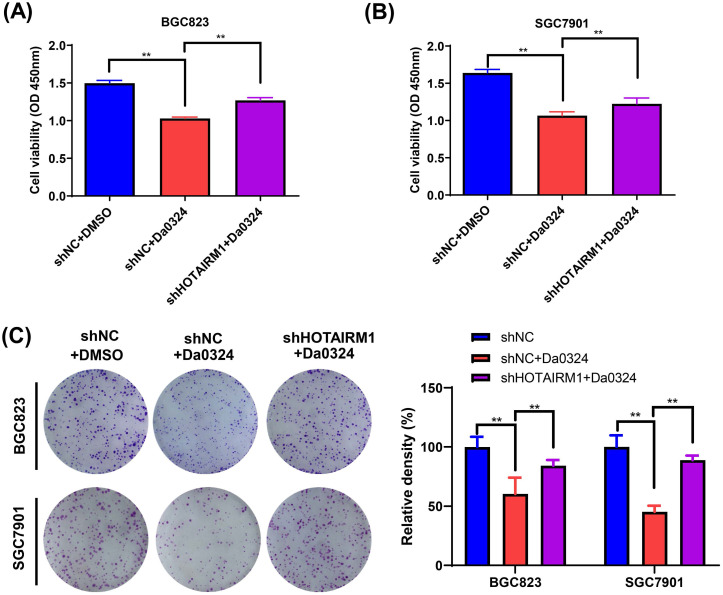
** Knockdown of HOTAIRM1 alleviates Da0324-induced cytotoxicity in gastric cancer cells. (A-B)** BGC823 (A) and SGC7901 (B) cells transduced with shNC or shHOTAIRM1 were exposed to 4 µM Da0324 for 48 h, and the cell viability was examined by the CCK-8 assay. **(C)** BGC823 and SGC7901 cells transduced with shNC or shHOTAIRM1 were exposed to Da0324 (1.5 µM) for 48 h and colony formation was assessed. All experiments were performed in triplicate, and results are presented as the mean ± SD. ^**^, *P* < 0.01.

We used HOTAIRM1-silenced BGC823 and SGC7901 cells to investigate the biological function of HOTAIRM1 in Da0324-induced cytotoxicity* in vitro*. As shown in **Figure 4A and 4B**, the results of CCK-8 assays showed that Da0324 treatment significantly reduced GC cell viability and the knockdown of HOTAIRM1 partially reversed the growth inhibition induced by Da0324. In addition, a similar pattern was observed in the plate colony formation assays (**Figure 4C**).

### HOTAIRM1 acts as a sponge for miR-29b-1-5p in GC cells

**Figure 5:**
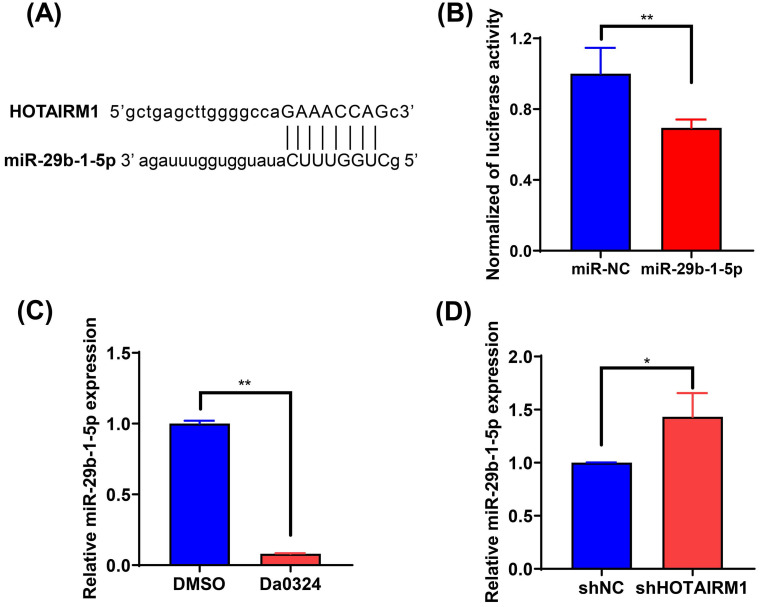
** HOTAIRM1 acts as a sponge for miR-29b-1-5p in gastric cancer. (A)** lncRNAMap was used to predict the binding site of miR-29b-1-5p in the HOTAIRM1 sequence. **(B)** Dual-luciferase assay was performed to verify the binding of miR-29b-1-5p to HOTAIRM1. **(C)** The relative expression level of miR-29b-1-5p was determined in SGC7901 cells treated with 4 µM Da0324 for 48 h. **(D)** qRT-PCR analysis of miR-29b-1-5p expression in SGC7901 cells transduced with lenti-shHOTAIRM1. All experiments were performed in triplicate, and results are presented as mean ± SD. ^*^, *P* < 0.05; ^**^, *P* < 0.01.

To investigate the effect of HOTAIRM1 on the expression of miRNAs, we used the online database lncRNAMap to predict the miRNAs that interacted with HOTAIRM1. Bioinformatics analysis indicated that miR-29b-1-5p shared complementary bonds in the HOTAIRM1 sequence (**Figure 5A**). To further validate the interaction between HOTAIRM1 and miR‐29b-1-5p, luciferase reporter constructs were generated. The results of the luciferase reporter gene assay showed that miR‐29b-1-5p mimic decreased the luciferase activity of the HOTAIRM1 luciferase reporter vector compared with the negative control (**Figure 5B**). Moreover, miR‐29b-1-5p expression was significantly decreased in SGC7901 cells with Da0324 treatment compared with untreated control cells (**Figure 5C**), whereas silencing of HOTAIRM1 expression increased miR‐29b-1-5p level in SGC7901 cells (**Figure 5D**). All these results indicate that Da0324 up-regulates the expression of HOTAIRM1 and HOTAIRM1 could sponge miR-29b-1-5p to suppress its expression.

### miR-29b-1-5p regulates Da0324-induced growth inhibition of GC cells

**Figure 6:**
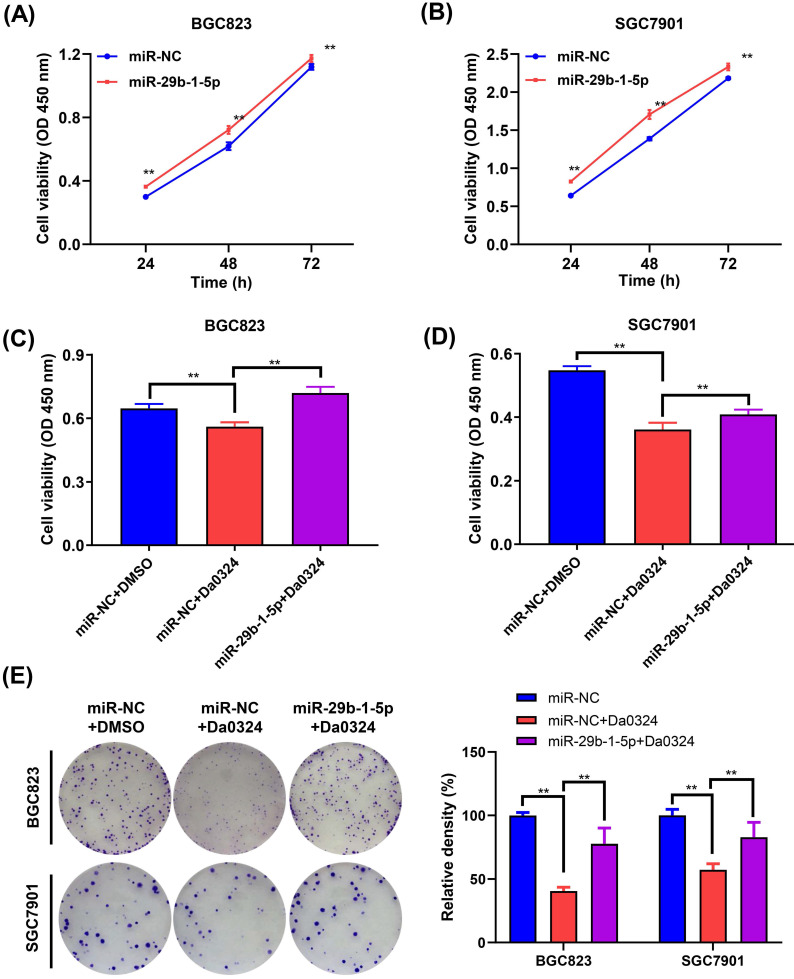
** miR-29b-1-5p regulates Da0324-induced cytotoxicity in gastric cancer cells. (A-B)** Cell proliferation was assessed by CCK-8 assays in BGC823 (A) and SGC7901 (B) cells transfected with miR-NC control or miR-29b-1-5p mimics. **(C-D)** Relative viability of BGC823 (C) and SGC7901 (D) cells pre-transfected with miR-29b-1-5p mimics was determined by CCK-8 assays after 24 h Da0324 (4 µM) treatment. **(E)** BGC823 an SGC7901 cells transfected with miR-29b-1-5p mimics were treated with Da0324 (1.5 µM) for 48 h, and then the clonogenic assay was used to detect the colony-forming ability of BGC823 and SGC7901 cells. Data are expressed as mean ± SD (n = 3). ^**^, *P* < 0.01.

Next, we examined the role of miR‐29b-1-5p in the growth of GC cells. BGC823 and SGC7901 cells were transfected with miR‐29b-1-5p mimics or the corresponding NC, and the CCK‐8 assay was performed to verify whether miR‐29b-1-5p was associated with GC cell growth. Compared with the NC group, overexpression of miR‐29b-1-5p increased cell growth (**Figure 6A and 6B**). To understand whether miR‐29b-1-5p is involved in Da0324 inhibiting the growth of GC cells, BGC823 and SGC7901 cells transfected with miR‐29b-1-5p mimics or negative control were treated with Da0324. As shown in **Figure 6C and 6D**, overexpression of miR‐29b-1-5p reversed the Da0324-induced growth inhibition in BGC823 and SGC7901 cells. Similarly, the results of clone formation assays showed that Da0324 significantly attenuated proliferation of BGC823 and SGC7901 cells; however, these effects could be reversed after transfection with miR‐29b-1-5p mimics (**Figure 6E**). These results suggest that Da0324 inhibits the growth of GC cells and may be involved in the regulation of miR‐29b-1-5p expression.

### PHLPP1 is regulated by the HOTAIRM1/miR-29b-1-5p axis in GC cells

**Figure 7:**
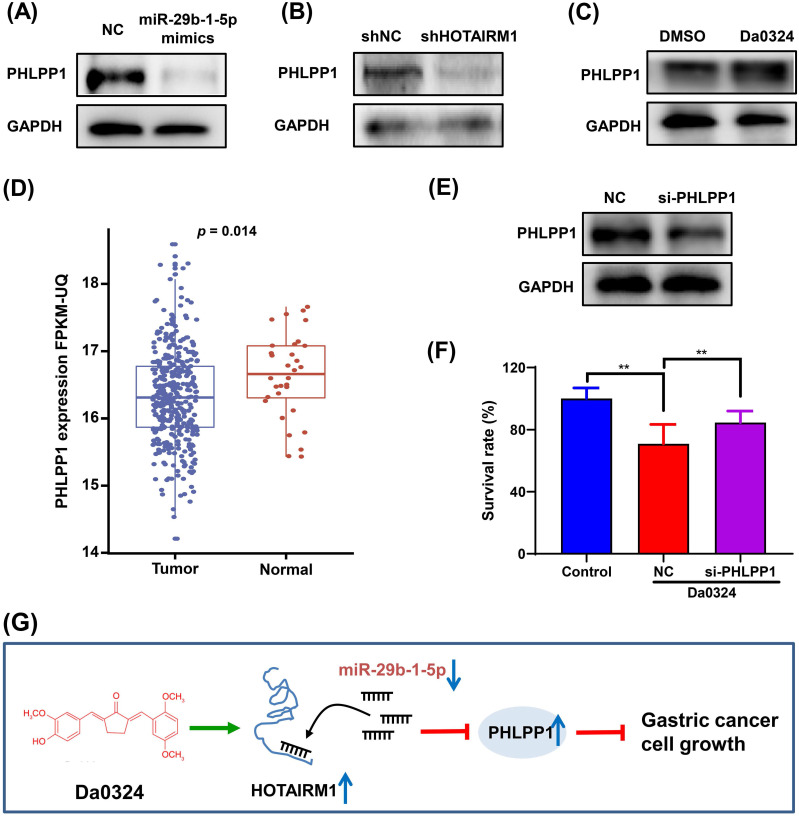
** PHLPP1 is regulated by the HOTAIRM1/miR-29b-1-5p axis in gastric cancer cells. (A)** Western blot analysis of PHLPP1 protein level in SGC7901 cells transfected with miR-29b-1-5p mimics. **(B)** Western blot analysis of PHLPP1 protein level in SGC7901 cells transfected with shHOTAIRM1. **(C)** SGC7901 cells were treated with Da0324 (4 µM) for 48 h, PHLPP1 protein expression was determined by Western blotting. **(D)** PHLPP1 expression in normal and gastric cancer samples based on TCGA data. **(E)** The knockdown efficiency of si-PHLPP1 was verified in SGC7901 cells by Western blotting. **(F)** Cell proliferation of SGC7901 cells transfected with si-PHLPP1 or negative control and treated with Da0324, according to the CCK-8 assay. Data are expressed as mean ± SD (n = 3). ^**^, *P* < 0.01. **(G)** Mechanistic model of Da0324-induced growth inhibition of gastric cancer cells.

PHLPP1 has been reported to be a target of miR-29b-1-5p 30. Therefore, we investigated the role of PHLPP1 in Da0324-induced growth inhibition of GC cells. Western blot analysis showed that overexpression of miR-29b-1-5p significantly inhibited the protein expression of PHLPP1 in SGC7901 (**Figure 7A**). Also, HOTAIRM1 silencing significantly suppressed the level of PHLPP1 protein (**Figure 7B**), whereas administration of Da0324 increased the protein expression of PHLPP1 (**Figure 7C**). These findings suggest that Da0324 up-regulates PHLPP1 expression by promoting HOTAIRM1 and suppressing miR-29b-1-5p. Moreover, PHLPP1 expression was significantly down-regulated in GC compared with normal samples based on TCGA data (**Figure 7D**). Knockdown of PHLPP1 significantly reversed the effect of Da0324 on GC cell growth (**Figure 7E and F**). These results suggest that Da0324 inhibits the growth of GC cells *via* the HOTAIRM1/miR-29b-1-5p/PHLPP1 axis (**Figure 7G**).

## Discussion

Interest in the use of natural compounds such as curcumin to treat cancer has increased. More and more research on curcumin has shown that it exerts anti-tumor effects by regulating various biological molecules such as cytokines, adhesion molecules, growth factors, and their receptors 31. Experimental studies with curcumin have shown that it can inhibit tumor initiation, proliferation, metastasis and invasion of GC cells 32. For example, curcumin inhibits proliferation and induces autophagy and apoptosis in GC cells by activating the P53 signaling pathway and inhibiting the PI3K signaling pathway 33. The poor bioavailability and pharmacological kinetics of curcumin hinder its therapeutic potential. In our previous studies, to improve the above shortcomings, we have developed novel analogs of curcumin Da0324, which showed significantly improved stability and anti-cancer activity in GC cells 2, 20. In this study, we further searched for the possible mechanisms that could provide new ideas to support its intriguing anti-cancer activity.

As potential anti-carcinogens, curcumin and its analogues have been explored for their potential to regulate lncRNAs 34. For example, curcumin treats gliomas by regulating the negative feedback loop of H19/miR-675/VDR 35. Furthermore, Yoshida et al. observed that curcumin reduced the resistance of pancreatic cancer cells to gemcitabine by inhibiting the expression of lncRNA PVT1 36. Here, we identified all statistically altered lncRNAs in GC cells induced by Da0324 with high-throughput sequencing. In particular, we found that HOTAIRM1 was up-regulated in GC cells by Da0324 treatment. HOTAIRM1 is specifically expressed in the myeloid lineage, and was initially identified as a myeloid-specific regulator of the HOXA gene family 37. Recent studies have shown that the function of HOTAIRM1 is different in different types of cancer. In leukemia 38, glioblastoma 39, 40, non-small cell lung cancer 41, 42 and thyroid cancer 43, the expression of HOTAIRM1 is upregulated and it functions as an oncogene. However, HOTAIRM1 is downregulated in the hepatocellular carcinoma 40, colorectal cancer 44, 45, ovarian cancer 46 and GC 28, and is absolutely a suppressor factor. Hai Hu et al reported that HOTAIRM1 inhibits cell progression by regulating miR-17-5p/PTEN axis in GC which is consistent with our results 28. The reason why HOTAIRM1 acts as a promoter or suppressor in opposite ways in different cancer is still unclear. However, it is not an occasional event. Many human genes have been found to be altered in very tissue-specific ways during tumorigenesis 47. Different tissues have a completely different microenvironment. Cancer cells are facing the different external pressures that cause proliferation regulators to exert selective functions 47. Here, we found that HOTAIRM1 transcription was activated by Da0324 in GC cells, suggesting that the anti-tumor effects of Da0324 might be caused by the up-regulation of HOTAIRM1. Our study showed that HOTAIRM1 silencing promoted GC cell proliferation whereas overexpression of HOTAIRM1 inhibited GC cell proliferation as detected by CCK-8 and colony formation assays. These results indicate that upregulation of HOTAIRM1 can inhibit the development of GC, which is consistent with the findings of Lu et al. 28. Moreover, functional assays showed that knockdown of HOTAIRM1 could attenuate the inhibitory effect of Da0324 on GC cell proliferation *in vitro*, suggesting that Da0324 exerts its anti-cancer effect at least in part *via* upregulation of HOTAIRM1.

lncRNAs affect gene regulation through a variety of mechanisms. One of the mechanisms is that they function as ceRNAs and regulate gene expression by sponging corresponding miRNAs 37, 48. In our study, subcellular localization experiments were performed to prove that the expression of HOTAIRM1 was significantly higher in the cytoplasmic fraction of GC cells, supporting the potential of HOTAIRM1 to function as ceRNA. In addition, bioinformatics analysis and luciferase reporter assay proved that miR-29b-1-5p was the downstream target of HOTAIRM1. Kim et al. demonstrated that miR-29b-1-5p, acting as a GC development promoter, regulated GC cell migration by targeting CREBZF 49. Additionally, it was reported that the lower expression of miR-29b-1-5p reduced the IC50 of human breast cancer parental cell line MCF-7 selected at 500 nM adriamycin, and the higher expression of miR-29b-1-5p weakened the effect of liposomal curcumin to Adriamycin-resistance 50. We found that Da0324 treatment down-regulated the expression of miR-29b-1-5p, while knockdown of HOTAIRM1 up-regulated miR-29b-1-5p expression. Also, the manipulation of the expression level of miR-29b-1-5p with a mimic promoted GC cells proliferation. In addition, overexpression of miR-29b-1-5p reversed the Da0324-induced growth inhibition in GC cells. These results indicate that Da0324 may suppress GC cell proliferation by up-regulating HOTAIRM1 expression that binds to miR-29b-1-5p, leading to down-regulation of miR-29b-1-5p.

PHLPP1 has been reported to be a direct target of miR-29b-1-5p 30. Knockdown of miR-29b-1-5p inhibited the migration of AGS cells, knockdown of PHLPP1 increased migration rates in Helicobacter pylori-treated AGS cells 30. Several research studies have shown that decreased or lost PHLPP1 expression has been detected in many cancers, such as prostate cancer, colorectal cancer, pancreatic cancer and glioblastoma 51-54. The low expression of PHLPP1 has been related to GC 55. In this study, we found that overexpression of miR-29b-1-5p or knockdown of HOTAIRM1 decreased the expression of PHLPP1, while Da0324 treatment increased the expression of PHLPP1 in GC cells. Knockdown of PHLPP1 significantly reversed the effect of Da0324 on GC cell growth inhibition, suggesting that the efficacy of Da0324 is partially dependent on increasing the level of PHLPP1. Taken together, these results suggest that the mechanism of anti-cell proliferation induced by Da0324 is achieved by regulating the HOTAIRM1/miR-29b-1-5p/PHLPP1 axis. Overall, our study elucidated a novel molecular mechanism of the anti-cancer activity of Da0324 in GC cells. HOTAIRM1 transcription was activated by Da0324 treatment in GC cells. Subsequently, HOTAIRM1 inhibited GC cell growth through promoting PHLPP1 expression by sponging miR-29b-1-5p. These data provide new insights into understanding the biological function and downstream regulatory network of Da0324.

## Supplementary Material

Supplementary figures.

## Abbreviations

CCK-8: Cell counting kit-8
ceRNAs: Competitive endogenous RNAs
DMEM: Dulbecco modified Eagle medium
GC: Gastric cancer
lncRNA: Long non-coding RNA
miRNAs: microRNAs
NC: Negative control
ncRNAs: Non-coding RNAs
PVDF: Polyvinylidene fluoride
qRT-PCR: Quantitative real-time PCR
SDS-PAGE: Sodium dodecyl sulfate-polyacrylamide gel electrophoresis
SD: Standard deviation
shRNA: Short hairpin RNA
siRNA: Small interference RNA
