# Supplementary material for: The Curcumin Analog Da0324 Inhibits the Proliferation of Gastric Cancer Cells via HOTAIRM1/miR-29b-1-5p/PHLPP1 Axis
Source: J Cancer. 2022 May 16;13(8):2644–55. doi: 10.7150/jca.69970 (PMC9174869; doi:10.7150/jca.69970)
Supplement: Supplementary file 1 — Supplementary figures. [file jcav13p2644s1.pdf]

Supplementary Data

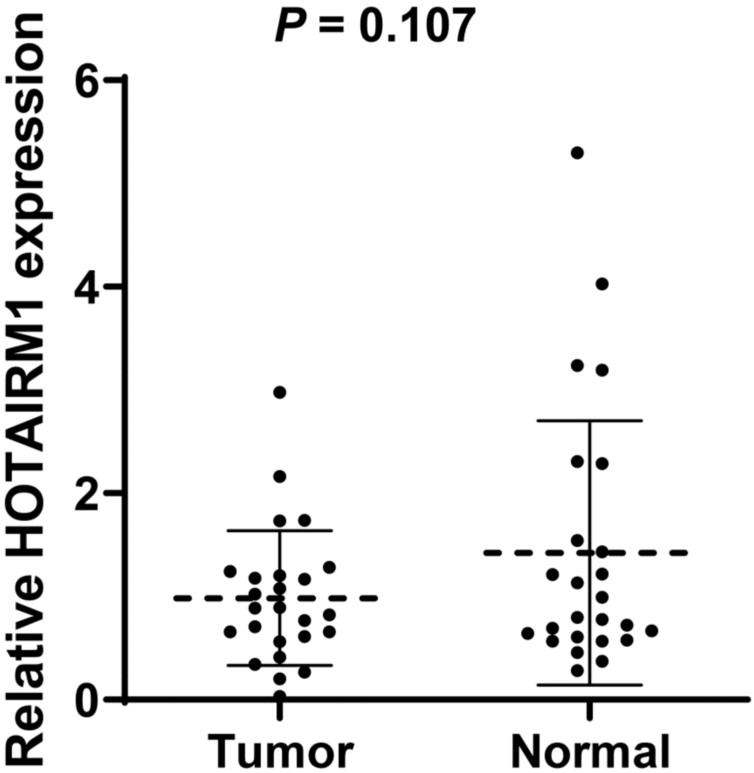

Figure S1 qRT-PCR analysis of HOTAIRM1 expression in gastric cancer and normal tissues (n =25).

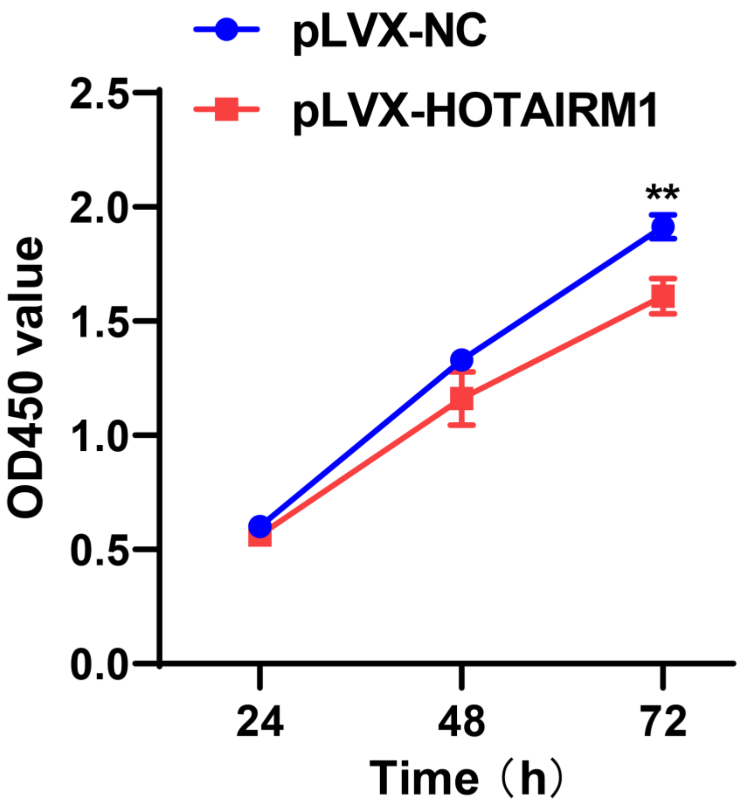

Figure S2 The CCK-8 assay results showing the effect of HOTAIRM1 overexpression on the proliferation of AGS cells.

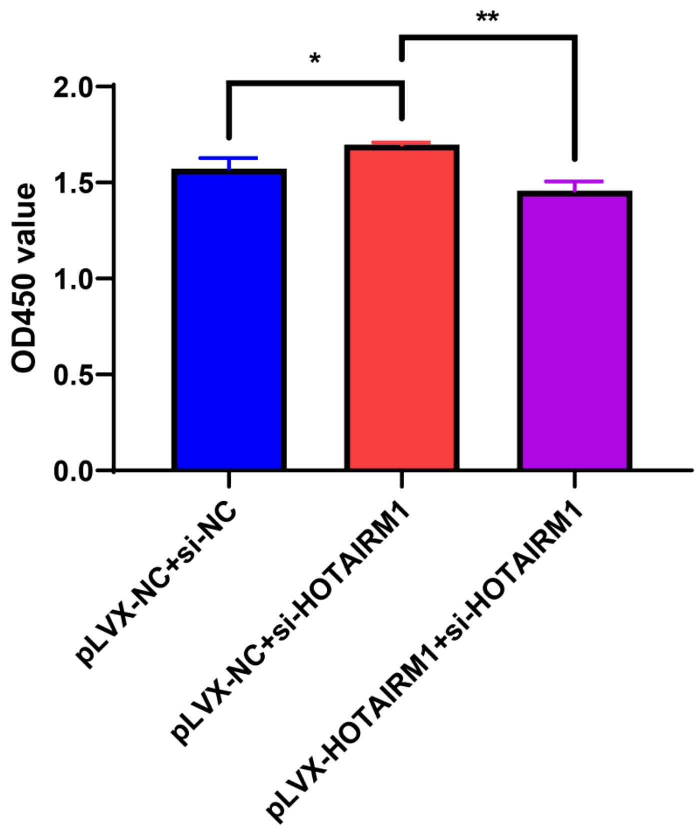

Figure S3 Cell proliferation was measured by CCK-8 assay. BGC832 cells were transfected with si-HOTAIRM1-1 and pLVX-HOTAIRM1 for 48 h.
